# Autoimmune Rheumatic Diseases Masquerading as Psychiatric Disorders: A Case Series

**DOI:** 10.31138/mjr.32.2.164

**Published:** 2021-06-30

**Authors:** Jayaprakash Russell Ravan, Sucheta Chatterjee, Pratima Singh, Debashis Maikap, Prasanta Padhan

**Affiliations:** 1 Department of Psychiatry, Kalinga Institute of Medical Sciences, KIIT University, Bhubaneswar, Odisha, India; 2 Department of Pulmonary Medicine, Kalinga Institute of Medical Sciences, KIIT University, Bhubaneswar, Odisha, India; 3 Department of Clinical Immunology and Rheumatology, Kalinga Institute of Medical Sciences, KIIT University, Bhubaneswar, Odisha, India

**Keywords:** Autoimmune, depression, psychosis, anxiety

## Abstract

**Background:**

Neurological involvement has been found in many autoimmune diseases, with psychiatric abnormalities such as anxiety, depression, psychosis, and cognitive dysfunction.

**Patients and Methods:**

Here, we describe a series of 5 consecutive cases of autoimmune diseases presenting with psychiatric symptoms as the predominant manifestation.

**Result:**

Our case series suggests that psychiatric symptoms, mainly depression, can be one of the presenting symptoms of several autoimmune diseases that often cause a significant delay in diagnosis.

**Conclusion:**

Any patient, particularly females, with a newly detected psychiatric disorder that responds poorly to medical management should be properly examined for underlying primary systemic autoimmune diseases.

## INTRODUCTION

The link between autoimmune diseases and psychiatric disorders gathers much attention from researchers. Research in the field of psychoneuroimmunology is still evolving, with many dimensions being investigated. The brain-immune system interaction is crucial in maintaining homeostasis and would help develop an understanding of various neuropsychiatric manifestations. A combination of host genetic factors and exposures to environmental triggers lead to the activation of the auto-immune pathway. This produces auto-antibodies and the dysregulation of T-cells and B-cells in a genetically susceptible person. Auto-antibodies such as anti-neuronal antibodies are implicated in the pathogenesis of the neuropsychiatric symptoms seen in many autoimmune diseases, especially Lupus. These autoantibodies can be found in the blood and cerebrospinal fluid (CSF) of patients with psychosis, and higher levels can be seen in acute relapse.^1^ Various epidemiological studies have found a general association between psychotic disorders and autoimmunity.^2^

Although neurologic manifestations can be seen in any autoimmune rheumatic disease (AIRD), the more common diseases like Lupus, primary Sjögren’s syndrome, multiple sclerosis, Celiac disease, and Grave’s disease are often associated with many neuropsychiatric symptoms. These symptoms range from anxiety and depression to psychosis.

Here, we describe 5 patients who initially presented with neuropsychiatric symptoms to the Psychiatric outpatient department with an underlying primary systemic autoimmune disease. Analysis of this case series affords insight into the immunological basis of psychiatric manifestations as well as an understanding of the rational approach, appropriate referral, and collaborative management of these and similar patients.

## CASE SERIES

### Case 1

A 46-year-old post-menopausal female was presented to us with symptoms of social anxiety, palpitations, difficulty speaking, and fear of choking on her food. She was found to have underlying mild depression, characterised with low mood and lethargy with disturbed sleep. She was given supportive psychotherapy with benzodiazepine and started on Amitriptyline 25mg/day. However, she complained of increased discomfort due to dry mouth. Her medication was changed to Sertraline 25mg/day and gradually increased to 150mg/day for six weeks, but the dryness of mouth continued. She was referred to the rheumatologist. On repeated enquiry, she told to have symptoms of dry mouth and dry eye. She also gave a history of arthralgia. On blood investigation, anti-Ro and La were found to be positive with positive anti-nuclear antibodies (ANA). Her anti-dsDNA/Smith was negative with normal complements. Her Schirmer’s test for dry eyes was positive. She was diagnosed with primary sjogren’s syndrome and started on low dose steroids and HCQs 200mg per day. Her symptoms improved after three months, and gradually her antidepressants were tapered and stopped over 1year. Currently, she is doing well with HCQs monotherapy.

### Case 2

38-year-old female patient presented to psychiatry OPD with complaints of low mood, anergy, anhedonia, crying spells, decreased social interaction, disturbed sleep, feeling dull at all times, loss of appetite with resultant significant weight loss, easy fatigability, and generalised body pain for nine months. She was diagnosed with severe depression without psychotic symptoms. She was started on sertraline 25mg/day and gradually increased to 150mg/day, however, showed inadequate response. Over a period of time, she was tried on multiple anti-depressants as she consulted various psychiatrists. However, she had partial or poor response in asthenia and easy fatigability. After ten months, she noticed tightening of the skin in face and extremity with shiny skin which was leathery to touch. She was referred to the rheumatologist. Upon detailed history, she had Raynaud’s phenomenon and symptoms suggestive of reflux esophagitis. She also revealed shortness of breath on physical exertion, but no cough or haemoptysis associated with it. Clinical diagnosis of diffuse systemic sclerosis was made, and Scl-70 was found to be positive in immunoblot assay of ENA (extractable nuclear antibody) profile. HRCT (High resolution computed tomography) of the thorax revealed NSIP (nonspecific interstitial pneumonia) pattern of interstitial lung disease (ILD). She was started on low dose corticosteroids and Mycophenolate, and antidepressants were continued. Gradually her psychiatric symptoms improved, and antidepressants were subsequently stopped in 6 months. She is currently on Mycophenolate mofetil 2grams per day in divided doses along with Tadalafil 20mg per day and Omeprazole 20mg per day.

### Case 3

36-year-old female presented to psychiatry OPD with complaints of low mood, anergia with easy fatigability, generalised weakness with body pain, and disturbed sleep for six months. She was diagnosed with severe depression without psychotic symptoms. She was started on Fluoxetine 20mg/day and gradually increased to 40mg/day, however showed inadequate response. Over a period of time, she was tried on Venlafaxine 150 mg/day and lithium 400 mg/day. However, she had partial or poor response in aspects of energy levels and easy fatigability. After a span of eight months, she complained of dry cough with exertional dyspnoea. Rheumatology consultation was sought. On examination, she had skin tightening of the face and extremities bilaterally, as well as Raynaud’s phenomenon. Diagnosis of systemic sclerosis was made. Her lung function study showed Forced Vital Capacity of 35%. On HRCT, the patient showed signs of interstitial lung disease (NSIP pattern). Despite being on cyclophosphamide pulse therapy for six months, her lung condition deteriorated over time, and she succumbed to respiratory failure within one year of diagnosis.

### Case 4

A 22-year-old female presented with symptoms of low mood, anergy, generalized weakness, crying spells, difficulties sleeping for past two months. She was diagnosed with moderate depression and started on escitalopram 10mg/day. After two months, she reported no improvement in symptoms but presented with irritability, anger outbursts, feeling fearful, and an inability to sleep. The diagnosis was revised to severe depression with psychotic symptoms, and low dose antipsychotic olanzapine 5mg/day was added. After two days, she developed oral ulcers on the palate with erythematous rash in a butterfly pattern on the cheeks with photosensitivity. She was referred to the rheumatologist for evaluation of Lupus. She had features of mucocutaneous and neuro-psychiatric symptoms of Lupus. On blood investigation, ANA (antinuclear antibody by IIF-Indirect immunofluorescence) and anti-dsDNA was found to be positive with low complement levels. Evaluation for the anti-phospholipid syndrome was negative. Her urine examination did not reveal proteinuria or any active sediment ruling out renal involvement. Her liver function, renal function and thyroid function tests were normal. Her brain imaging (MRI brain) and CSF studies for protein, glucose and cell counts were normal. She was started on 1mg/kg predniso-lone per day, and hydroxychloroquine 200mg per day. Mycophenolate 2gm/day was added as a steroid sparing agent. Her neuropsychiatric symptoms improved, and her antipsychotic medications were slowly tapered and stopped. She has been doing well for the last 1 year.

### Case 5

34-year-old female patient presents to psychiatry OPD with symptoms of low mood, easy fatigability, generalised weakness, and body aches with sleep disturbance for six months. She was diagnosed with moderate depression with fibromyalgia. She was started on low dose Desvenlafaxine at 50mg/day gradually increased to 100mg/day over a period of time. Even though the patient reported improvement of mood symptoms, she consistently complained of fatigue. She developed shortness of breath and cough. On evaluation, she had bilateral hilar lymphadenopathy on CT thorax and elevated serum Angiotensin-converting enzyme (ACE) levels. MRI brain was normal, which excluded neurosarcoidosis. Hence, a diagnosis of sarcoidosis with lung involvement was made and started on prednisolone 40mg per day. Weekly methotrexate was added subsequently as a steroid sparing agent. Her symptoms improved. Antidepressants are tapered and stopped over a period of 1 year.

## DISCUSSION

Neuropsychiatric symptoms are more common in auto-immune disease than previously thought, and can mimic systemic autoimmune rheumatic diseases. This is rarely reported in the literature.

Psychoneuroimmunology is the cross-talk between the neuroendocrine system and immune system. This field is imperative for proper physical and mental health as well as to maintain homeostasis. Neural regulation of immune responses is accomplished systemically by hormones, regionally by innervation, and locally by neurotransmitters. There is evidence for rich neural innervation in lymphoid tissue^3^ with an expression of receptors on lymphocytes for various neurotransmitters besides acetylcholine and norepinephrine receptors. The parasympathetic nervous system modulates classic immune responses via acetylcholine through the vagus nerve. The sympathetic nervous system can alter the TH1/TH2balance through stimulation of the beta-adrenergic receptor on lymphocytes. Neurons may inactivate T cells and microglia through both contact-dependent and -independent mechanisms. Glial cells (astrocytes and microglia) in the brain are activated through various soluble factors secreted by peripheral immune cells such as cytokines and chemokines that penetrate the blood brain barrier. Excessive or prolonged glial activation may result in neuro-inflammation and a more severe and chronic neuronal damage through a mechanism known as excitotoxicity. This will predispose to various neuropsychiatric manifestations, such as depression, cognitive impairment, and psychosis. Moreover, various immunosuppressive neuropeptides and neurotransmitters, including vasoactive intestinal peptide (VIP), norepinephrine (NE), and γ-aminobutyric acid (GABA) are present in neurons. *In vivo* murine models of Parkinson’s Disease, acute brain injury, neuroinflammation and cerebral ischemic show VIP is a ubiquitous neuropeptide with neuroprotective properties through its inhibition of proinflammatory mediators, such as IL-6, tumour necrosis factor (TNFα), IL-12, and nitric oxide (NO).^4^ Norepinephrine acts as both a hormone and neurotransmitter that dampens cellular immunity and suppresses neuro-inflammation in the brain.^5^ Similarly, GABA is a inhibitory neurotransmitter in the brain that accentuates lipopolysaccharides (LPS) induced IL-6 and IL-10 production by microglia.^6^ affecting the entry of pathogenic T lymphocytes into the brain and T-cell proliferation. Systemic or intrathecal administration of IL1β or TNFα induces signs of behaviour resulting from sickness, such as decreased motor activity, social withdrawal, altered cognition, and fatigue.^7^

The connecting links between autoimmune disorders and mental illness could be the presence of antineuronal antibodies and dysregulation in B and T cell homeostasis. Autoimmune disorders and depression correlate reciprocally to each other in such a way that either can be cause or effect. Majority of patients with autoimmune rheumatic diseases subsequently develop psychiatric symptoms such as depression and anxiety as a result of prolonged illness, excessive pain, and sleep disturbance. Depression can change the pain perception, which is linked to fatigue and sleep disorders. Medical therapy such as anti-inflammatory and immunomodulators results in alleviation of these symptoms in many subjects. According to one study, the incidence of depression in rheumatoid arthritis (RA) and lupus were 74% and 64% respectively.^8^ Psychiatric disorder can also result from medications used to treat autoimmune diseases, like interferon type I in multiple sclerosis or corticosteroids in lupus.^9^

Depression can be of 2 types - endogenous and reactive. Familial or endogenous depression results from responses to stress in early life. Reactive depression is secondary to a medical illness that causes physical stress to neuro-homeostasis, through a cascade of metabolic or inflammatory processes causing alterations in the mood neurotransmitters.^8^ Our cases did not have reactive or familial depression.

In Primary Sjögren’s syndrome (pSS), neuropsychiatric manifestations have been reported in up to 80% of adults; and often, it precedes the diagnosis in up to 50–80% cases. Anxiety, depression, and cognitive dysfunction are commonly seen in pSS.^10^ Neurological manifestations in pSS are more commonly associated with SSA than SSB antibodies.^11^ In our 1st case, primary Sjögren’s syndrome initially presented with depressive symptoms unresponsive to standard antidepressants. Sicca symptoms were eventually unmasked.

Psychiatric symptoms such as anxiety, depression, obsessive-compulsiveness, somatisation, and feelings of guilt were reported by the majority of patients with scleroderma.^12^ Depression in systemic sclerosis in various clinical studies has been found to be 65% or higher.^13^ Two of our cases (2 and 3) with systemic sclerosis initially presented to us with significant depressive symptoms. Systemic Lupus Erythematosus (SLE) is another autoimmune disease known to have neuropsychiatric symptoms such as anxiety, depression, cognitive dysfunction, and psychosis with a prevalence of 21–95% of patients. These symptoms can occur within the first year of diagnosis or can rarely even be the primary presenting symptom. Only 13–38% of psychiatric abnormalities are directly related to SLE, whereas the remaining attributed to treatment complications like glucocorticoid use.^14^ About one-fourth of lupus patients have severe psychiatric disorders.^15^ Our 4^th^ case had neurolupus with neuropsychiatric symptoms that were eventually managed successfully with immunosuppressants.

Sarcoidosis is associated with fatigue, sleepiness, fibromyalgia, and a high rate of psychiatric comorbidity (6.3%).^16,17^ A high degree of suspicion is required for the diagnosis of organic disease, such as sarcoidosis. As an example, our last case had psychiatric manifestations as the predominant feature.

This case series describes 5 cases who presented to psychiatry OPD with complaints of depression and/or anxiety that were later diagnosed as primary immunological entities. The patients were all female and complained of chronic fatigue with unexplained persistent somatic symptoms that were poorly responsive to adequate doses of antidepressants given over an adequate period of time with good compliance.

## CONCLUSION

Autoimmune diseases should be considered in the differential diagnosis of any patients, particularly females, with a new psychiatric disorder that are responding poorly to antidepressants and/or antipsychotics. The above case series highlights how detailed history and clinical examination are essential in the diagnosis of underlying primary systemic autoimmune diseases.

## References

[1] Jeppesen R Benros ME. Autoimmune diseases and psychotic disorders. Front Psychiatry 2019;10:131. 30949074 10.3389/fpsyt.2019.00131 PMC6435494

[2] Chen S-J Chao Y-L Chen C-Y Chang C-M Wu EC-H Wu C-S Prevalence of autoimmune diseases in in-patients with schizophrenia: nationwide population-based study. Br J Psychiatry 2012;200:374–80. 22442099 10.1192/bjp.bp.111.092098

[3] Pongratz G Straub RH. The sympathetic nervous response in inflammation. Arthritis Res Ther 2014 12 1;16(6):504. 25789375 10.1186/s13075-014-0504-2 PMC4396833

[4] Delgado M Varela N Gonzalez-Rey E. Vasoactive intestinal peptide protects against beta-amyloid-induced neurodegeneration by inhibiting microglia activation at multiple levels. GLIA 2008;56:1091–103. 18442091 10.1002/glia.20681

[5] Heneka MT O’Banion MK. Inflammatory processes in Alzheimer’s disease. J Neuroimmunol 2007;184:69–91. 17222916 10.1016/j.jneuroim.2006.11.017

[6] Kuhn SA van Landeghem FK Zacharias R Farber K Rappert A Pavlovic S ET AL. Microglia express GABA(B) receptors to modulate interleukin release. Mol Cell Neurosci 2004;25:312–22. 15019947 10.1016/j.mcn.2003.10.023

[7] Miyake S. Mind over cytokines: Crosstalk and regulation between the neuroendocrine and immune system.

[8] Arias S Fonsalía V Asteggiante N Bartesaghi V. Systemic auto-immune diseases and depressive disorders. Reumatología Clínica (English Edition) 2011 11 1;7(6):389–91. 22078697 10.1016/j.reuma.2011.04.010

[9] Pryce CR Fontana A. Depression in autoimmune diseases. InInflammation-associated depression: Evidence, Mechanisms and Implications 2016 (pp. 139–154). Springer, Cham.

[10] Hammett EK Fernandez-Carbonell C Crayne C Boneparth A Cron RQ Radhakrishna SM. Adolescent Sjogren’s syndrome presenting as psychosis: a case series. Pediatric Rheumatol 2020 12 1;18(1):15. 10.1186/s12969-020-0412-8 PMC7014743 32046763

[11] Alexander EL Ranzenbach MR Kumar AJ Kozachuk WE Rosenbaum AE Patronas N Anti-Ro (SS-A) autoantibodies in central nervous system disease associated with Sjögren’s syndrome (CNS-SS): clinical, neuroimaging, and angiographic correlates. Neurology 1994;44(5):899–908. 8190294 10.1212/wnl.44.5.899

[12] Angelopoulos NV Drosos AA Moutsopoulos HM. Psychiatric symptoms associated with scleroderma. Psychother Psychosom 2001;70(3):145–50. 11340416 10.1159/000056240

[13] Thombs BD Taillefer SS Hudson M Baron M. Depression in patients with systemic sclerosis: A systematic review of the evidence. Arthritis Rheum 2007;57:1089–97. 17665491 10.1002/art.22910

[14] Tay SH Mak A. Diagnosing and attributing neuropsychiatric events to systemic lupus erythematosus: time to untie the Gordian knot? Rheumatology 2017 4 1;56(Suppl_1):i14–23. 27744358 10.1093/rheumatology/kew338

[15] Estes D Christian D: The natural history of systemic lupus erythematosus by prospective analysis. Medicine 1971;50:85–95. 4109481 10.1097/00005792-197103000-00001

[16] Goracci A Fagiolini A Martinucci M Calossi S Rossi S Santomauro T Quality of life, anxiety and depression in sarcoidosis. Gen Hosp Psychiatry 2008 9 1;30(5):441–5. 18774427 10.1016/j.genhosppsych.2008.04.010

[17] Bosse-Henck A Koch R Wirtz H Hinz A. Fatigue and excessive daytime sleepiness in sarcoidosis: prevalence, predictors, and relationships between the two symptoms. Respiration 2017;94(2):186–97. 28609770 10.1159/000477352

